# Perception of E-Resources on the Learning Process among Students in the College of Health Sciences in King Saud University, Saudi Arabia, during the (COVID-19) Outbreak

**DOI:** 10.3390/healthcare10010040

**Published:** 2021-12-26

**Authors:** Reham AlJasser, Lina Alolyet, Daniyah Alsuhaibani, Sarah Albalawi, Md. Dilshad Manzar, Abdulrhman Albougami

**Affiliations:** 1 Department of Periodontics and Community Dentistry, Dental College, King Saud University, Riyadh 11545, Saudi Arabia; 2 Department of General Dentistry, Dental College, King Saud University, Riyadh 11545, Saudi Arabia; Linaalolayet@hotmail.com (L.A.); Danzalsuhaibani@Gmail.com (D.A.); Albalawisarah@gmail.com (S.A.); 3 Department of Nursing, College of Applied Medical Sciences, Majmaah University, AlMajmaah 11952, Saudi Arabia; md.dilshadmanzar@gmail.com (M.D.M.); a.albougami@mu.edu.sa (A.A.)

**Keywords:** e-resources, e-learning, credibility, academic performance, health sciences education

## Abstract

Aim: to assess the impact of e-learning through different e-resources among health sciences students. Methodology: A cross-sectional design was conducted among health science students (*n* = 211; 134 female and 77 male) at King Saud University, Saudi Arabia. The data was collected using a previously used structured questionnaire to assess the impact of e-resources on learning. Results: The four most frequently used e-resources were: Zoom (38%), YouTube (31%), Google applications (29%), and Blackboard (27%). More than one-third of the students (35%) reportedly used e-resources for three or more hours daily. The majority of the students (55.9%) recognized a gender-related and age-related difference among faculty members in terms of e-resources usage. The majority of the students (58.2%) believe that online resources recommended by faculty members were credible. The majority of students believed that their academic performance was primarily influenced by these features of the e-resources: organization/logic of the content (64.5%), the credibility of the video (64.5%), and up to date “look and feel” of the video (60.6%). The study identified the most frequently used e-resources, gender, and age-related differences in faculty members’ use of e-resources, students’ overwhelming reliance on faculty feedback regarding the credibility of e-resources, and three most important characteristics (organization, credibility, and updated status) of e-resources. Conclusion: e-learning resources had a significant impact on participating students’ education as they were used very frequently during their health sciences’ courses.

## 1. Introduction

Recently, the outbreak of the pandemic situation due to Coronavirus (COVID-19) had led to massive loss of human life worldwide. The result of this massive loss spurred economic and social disruption. Therefore, it was recommended to limit social gathering and apply social distancing with increased precaution protocols to control this virus’s transmission. This recommendation was also applied globally at different levels of all educational systems as physical classes were stopped.

UNESCO estimates suggested that over 1.5 billion learners were affected during this period in the education system [1,2,3]. Therefore, alternatives have immediately been investigated and gathered to resume teaching and learning at different levels of education throughout the world.

Many educational institutes took the initiative to transfer traditional onsite learning to online education. This initiative was done in order to maintain the safety of their students and to fulfill their basic needs for education through distance learning.

Distance education, also known as distance learning, is defined as the education of students who may not always be physically present at a school [1,2]. Traditionally, this usually involved correspondence courses wherein the student corresponded with the school via mail, and today it usually involves online education.

Technological innovation has not only impacted social change in recent years but has been the prime driver of educational transformation [4,5,6,7]. There has been a growing interest in using Internet-based learning by universities over the past decade to supplement or replace traditional learning [8]. The development of new technologies marks the growth of the internet. E-learning is the use of internet-based resources in education. Internet-based learning for health professional education is increasing [9]. It offers advantages over traditional learning approaches, enables learning to be completed conveniently for the user, and improves accessibility, especially where facilities are geographically disparate [9,10]. It can also deliver a broad array of solutions that enhance knowledge and performance [8], increase accessibility to education, and improve self-efficacy [11] and clinical skills.

This results in improving practitioners’ capabilities [12], cost-effectiveness, learner flexibility [7], satisfaction and promotion of student-to-student and student-to-instructor interactions [13]. Therefore, it is of utmost importance to integrate e-learning to acquire knowledge in the study of health sciences.

Some barriers can affect the development and implementation of online learning in education, such as poor technical skills, inadequate infrastructure, absence of institutional strategies and support, and negative attitudes of stakeholders [14,15,16]. On the other hand, the practical nature of health science education demands direct contact between students, instructors, and patients [17]. Therefore, traditional teaching methods in health sciences are essential.

The use of digital devices in the college of health sciences for teaching and learning purposes has been widely accepted in universities [18]. As a result of this development, it has become apparent in recent years that Internet-based learning or electronic learning (E-learning) has increased its attraction to students at large [19]. E-learning has recently been proposed as a primary complementary tool to improve medical and dental education [20], which has been defined as learning while “utilizing electronic technologies to access educational curriculum outside of a traditional classroom” [21,22]. These interactive teaching strategies have enhanced students’ focus, amplified their attention, and maximized their long-term knowledge retention [20]. Therefore, most higher education institutions classify online learning as crucial for their educational strategy [23].

Electronic and virtual applications and sources can be compelling and entertaining in several educational fields. However, this can be very challenging in terms of application, especially in health science education which focuses on proper care, prevents the spread of diseases, and improves the lives of every patient to ensure the longevity of life or improvement in the life expectancy of individuals.

This field should provide students with technical skills, proper health care competencies, and various opportunities to obtain the knowledge needed for growth and development in the health sector [1]. It also aims to improve physical, mental, emotional, and social health by increasing their knowledge and influencing their attitudes by caring for their well-being [1,3].

King Saud University was one of the first universities in Saudi Arabia to transfer courses from traditional onsite to online education. Lectures were mainly given through live webinars to ensure proper interaction between the lecturer and the students. Assignments, quizzes, and exams were conducted through the BlackBoard website, which was previously activated during traditional education as a supportive tool for the educational process of the students. After the adoption of the online education system, the website became the backbone of the educational process.

With the ongoing spread of the coronavirus, online learning resources have become increasingly essential to ensure uninterrupted educational delivery to isolated students. This opportunity has expanded the learning offering beyond the limitations of the traditional methods. The literature revealed that learning technology has positively supported the health sciences curriculum [20]. Hence, it is crucial to evaluate its proper applicability in each specific education field and make a positive adjustment for maximum learning experience.

This study aims to assess the impact of e-learning through different e-resources among health sciences students attending King Saud University.

The objectives of the present study are as follows:

1. To understand the most prominent e-resources used among health sciences students attending King Saud University.
2. To assess the relationship between the age of faculty members related to given courses through e-resources and their use of these facilities.
3. To assess the relationship between the gender of faculty members related to given courses through e-resources and their use of these facilities.
4. To examine various e-resources and evaluate the effect of various sources of E-learning on health sciences students’ ability to comprehend academic topics.

Therefore, the Null hypothesis is that there is no positive impact of e-learning through different e-resources among health sciences students attending King Saud University.

The Alternative Hypothesis is that there is a positive impact of e-learning through different e-resources among health sciences students attending King Saud University

## 2. Materials and Methods

### 2.1. Ethical Considerations

Institutional review board approval was obtained from King Saud University, Riyadh, Saudi Arabia (E-20-5052). Informed consent was required for the participants to proceed to answer the questionnaire. Therefore responses without the participants’ consent were not recorded. The purpose and objectives of this study were explained, and the participants were informed that the information obtained was to be used for research purposes only, and the outcome would be presented in anonymous charts, figures, and tables.

### 2.2. Setting and Application

The survey was offered to undergraduate medical sciences students which included medical, dental, pharmacy, applied medical sciences, and nursing students from both female and male sections. An online survey was used, with all participants anonymously completing the survey at an opportune place and time for them.

A web-based survey with a link provided was distributed to participants through an e-mail and an invitation through social media platforms. Participants used a device and a browser of their choice and convenience. Investigators cannot identify participating students, and the survey was completely anonymous. Students were informed that they had the right to discontinue the study at any point in time without any consequences. The participation was sought to be voluntary, and the confidentiality of responses was maintained.

The proposed survey did not take more than five minutes to complete for the majority.

### 2.3. Sample Size Determination

The study focused on undergraduate students attending colleges of health sciences in King Saud University, Saudi Arabia. The sample size was determined by G Power software (Hinnerup, Denmark). The confidence level was set at 95%. The power level was set at 80% with a moderate effect size and a final sample size of 180 students. However, a larger sample was recruited to avoid the possibility of a low response rate that could affect the sample size. A final sample of 211 students was recruited using purposive sampling because the goal of this study focused on students in the college of health sciences [24].

### 2.4. Instrument to Be Used

A modified version was used after obtaining permission of the primary author of the survey for Student’s Perception of the Impact of E-Learning on Dental Education [14].

The survey was comprised of 14 questions, including seven multiple-choice questions, two fill-in-the-blank questions, two open-ended questions, and three Likert scale questions. The purpose of the two open-ended questions was to allow students to share the applications they used during their dental education, their perceived impact on their academic achievement, and their opinion of online education.

The first part contains questions related to demographic information.

The second part contains questions related to students’ use of E-learning resources (e-resources) and their perceptions of these resources.

The third part contained questions that asked students to mention the top three e-resources used for academic purposes. The questions explored several factors influencing the use of these e-resources which included the following: the time spent on these resources, the credibility of the e-resources recommended by faculty members, the influence of certain factors regarding e-resources on students’ academic achievement, the effect of e-resources on students’ ability to understand academic topics, students’ observation of faculty members integration of e-resources in their courses and its relationship with the faculty members age, the relationship that the students observed between the faculty member’s age and their dependence on social media for communication, the students’ attendance preference, and finally the effects of e-resources on students’ academic performance.

The modified version was sent to survey experts in the dental field to receive feedback and opinion about its clarity and easiness and recommendations for further adjustments. As a second step, a small sample of 20 students were chosen to pre-test the final version. This step showed that the questionnaire needed to become shorter and more straightforward. The participants also offered some amendments to the questionnaire which were considered and noted. The questionnaire was finalized after an in-depth discussion among the authors. The modified version was administered to a sample of 50 students in a pilot study. K value for the inter-participants’ agreement was calculated to as 0.91, indicating an “almost perfect agreement” according to Cohen [25].

### 2.5. Statistical Analysis

Descriptive Kolmogorov–Smirnov, and Shapiro–Wilks tests were applied to check the normal data distribution. The data was entered and analyzed using SPSS 24.0 version statistical software (IBM Inc., Chicago, IL, USA). Findings were presented through frequencies, percentages, mean, and standard deviation values. The following elements were evaluated: participants’ demographic characteristics, most frequently used electronic resources/applications by students, the average duration of daily electronic resources/applications used for academic performance, students’ observations of incorporation of e-learning by faculty members and the age of faculty members, and the relationship between faculty members’ dependence on social media for communication and their age.

## 3. Results

### 3.1. Participants’ Characteristics

In this study, health science students (*n* = 211; 134 females and 77 males) with a mean age of 21 years ± 3 years participated. About two-thirds of the participating students were enrolled in dentistry and the college of allied health sciences (Table 1). The majority of the study sample comprised of female students (63.5%).

### 3.2. Most Frequently Used E-Resources

Table 2 presents the descriptive summary of the item that required students to record their three most frequently used e-resources. Regarding preferred resources perceived by students to improve their academic performance, 16 different e-resources were identified. The four most frequently used were: Zoom (38%), followed by YouTube (31%), Google applications (29%), and Blackboard (27%) (Table 2). E.E.E., Saudi Digital Library, and Dropbox were the three least used electronics resources, with all the three being reported to be used by less than 1% of the participating students. More than one-third of the students identified ‘others’ as one of the resources.

### 3.3. Electronic Resources: Daily Use by Students, Gender and Age Pattern among Faculty Members

About 65 respondents (30.8%) used e-resources for two to three hours every day (Table 3). Most of the participating students reported either no gender-related difference or ‘I do not know’ in incorporating e-resources by faculty members (74%). However, the biggest group of students recorded that faculty members who more prevalently used e-resources were under 50 years of age (Table 4). Similarly, almost half of the students replied that there was a relationship between faculty members’ dependence on social media for communication and their age because it was more commonly seen in those under 50 years of age (Table 5).

### 3.4. Online Applications/Animations: Student’s Perceived Academic Performance and Reliance on Faculty Recommendations

**Figure 1:**
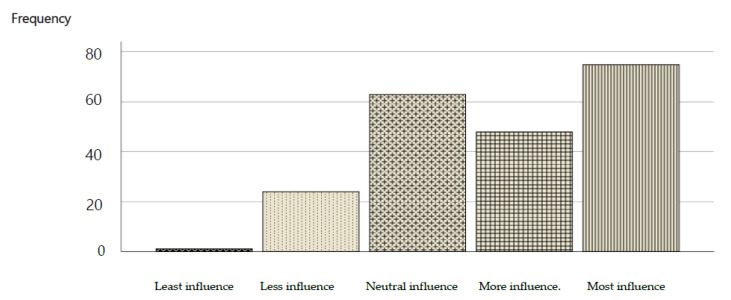
The “level of credibility” is given by students to e-resources recommended by faculty members.

Students regarded e-resources recommended by faculty members with a high level of credibility; this is indicated by a majority (58.2%) replying that they were greatly influenced by teachers’ feedback on such matters (Figure 1). Organization/logic of the content, credibility of the video, and up-to-date “look and feel” of the video were the three most influential factors on the students’ perceived academic performance with 64.5%, 64.5%, and 60.6%, respectively. While online presentation under 15 min was perceived to be least influential in academic performance, most students (55.9%) recorded a response of ‘neutral’ or ‘least influence’ for this factor (Table 6).

## 4. Discussion

The present study’s results highlighted that e-learning resources significantly impacted education during the coronavirus pandemic (COVID-19) outbreak when used by health sciences students at King Saud University.

Findings revealed that the most frequently used e-resources were Zoom, followed by YouTube, Google applications, and Blackboard. This sequence in preference could be attributed to the increasing urge for a videoconferencing technology to augment online learning, which unfolded at the pandemic’s peak when people were adjusting to the new normal. Zoom is one of the most helpful resources to enhance effective and synchronous e-learning since it allows visual interaction between the students and instructors. It was established that Zoom could accommodate 1000 participants in one meeting. This application can be downloaded and used for free (Ismawati, Iis, et al. 2021, Wibawanto, 2020) as cited by Kasman et al. [24,25] One could infer that due to its ability to accommodate a large number of participants, its simple interface and low cost explains the dominance of this e-resource compared to other applications.

In contrast, this result was supported by the findings from a previous study [14], we discovered that the second most used e-resource is YouTube. Before the pandemic, YouTube was globally the go-to site for virtual learning. However, recent authors [26,27] established that the COVID-19 outbreak pushed all universities to incorporate the use of video conferencing technology (V.C.T.) to supplement learning management systems for e-learning, including YouTube.

When the students were asked about the duration spent on e-resources, most of the respondents reported using e-resources for at least 3 h at a time. This relatively long duration can be explained by the nature of health sciences lectures and seminars, which usually require a significant amount of time.

Regarding the observed relationship between using e-resources and faculty members’ age, the majority of the respondents reported that the faculty members who prevalently used e-resources were more likely to be under the age of 50 years old. This may be due to the fact that the majority of this age category have been more exposed to recent technologies and more enthusiastic than the older age category. This result coincides with the results of the research work carried out by Turkyilmaz et al. [14], in which they discovered that faculty members that frequently used e-resources were under 50 years of age. They are more likely to have been exposed to technology in their education and early career stages. Therefore, they may be more inclined to use technology in learning settings and communication. Several investigators discovered that only a few faculty members use advanced online learning tools.

Faculty members hesitated to shift their teaching style to e-learning due to several reasons which included: low perceived benefit, difficulty in using these online resources, frequency of students’ usage, and the time required to invest in the process [24]. However, even faculty members over 50 years of age were compelled to use the e-resources in a limited fashion. This observation can also be explained by the variation of exposure to e-resources by different generations and the ubiquity of computers and the internet in modern academic environments. Moreover, this trend will keep increasing as more educators bring technology-based activities into the classrooms [14].

Furthermore, when faculty members’ gender was observed, results revealed that about 44% of the students did not perceive a gender-related pattern regarding incorporating e-resources. These findings were supported by a recent study where authors also discovered that the students in their study observed no gender-related pattern in incorporating distance learning [14].

Concerning students’ perceived academic performance and reliance on faculty members’ recommendations, the results indicated that most students viewed online applications recommended by faculty members with a high level of credibility. It was discovered that the organization/logic of the content, credibility and up-to-date “look and feel” of the video were the three most influential factors on the students’ perceived academic performance. These findings were contrary to the results of previous studies that reported the e-learning system’s efficacy on organization and attractiveness of the course content [12,13].

Several limitations have been observed in the present study, including statistical bias, as samples were not evenly distributed. Half of the sample size predominantly included dental students, while the other half contained medicine, nursing, pharmacy, and applied medical sciences. The present study is a cross-sectional study within one university which may limit the inference of the findings to other regions of the country or the world. Likewise, the study did not evaluate the students’ actual academic performance but rather their perceived performance, which may be subjective rather than objective. It should be highlighted that present findings were based only on surveys without structured interviews with faculty members. Therefore, future studies with a well-controlled and improved methodology are recommended to confirm present study findings.

In summary, the findings of this study helped understand the perception of health science students in adopting E-learning resources, the effect of E-learning on their ability to comprehend academic topics, and its influence on their academic performance.

## 5. Conclusions

E-learning resources were frequently used during the present study and they had significant impact on the participating health science students’ education. Most e-resources used were Zoom, followed by YouTube, Google applications, and Blackboard. The faculty members’ age was a significant factor affecting their use and reliance on e-resources. Organization, credibility, and updated status of e-resources were also significant contributors to health sciences students’ academic performance. In conclusion, incorporation of e-learning resources training and application in the schools’ curriculum is essential to improve health sciences students’ and faculty members’ distance learning experience and outcomes.

## Figures and Tables

**Table 1:** Participants’ characteristics.

Characteristics	Frequency	Percentage
Specialty		
College of Dentistry	111	52.6
College of Medicine	24	11.4
College of Pharmacy	17	8.1
College of Applied Medical Sciences	32	15.2
College of Nursing	14	6.6
Prince Sultan Bin Abdul-Aziz College for Emergency Medical Services	13	6.2
Age		
18–20	70	33.2
21–23	125	59.2
24 and more	16	7.6
Gender		
Male	77	36.5
Female	134	63.5

**Table 2:** Frequency distribution of students’ most frequently used electronic resources/applications in decreasing order.

Electronic Resources/Applications	Frequency	Percentage
Zoom	81	38.4
YouTube	66	31.3
Google applications	62	29.4
Blackboard	57	27.0
Notability	48	22.7
Microsoft Office applications	18	8.5
Telegram	12	5.7
Twitter	8	3.8
Adobe	7	3.3
WhatsApp	7	3.3
Flashcard applications	6	2.8
Instagram	4	1.9
Dropbox	1	0.5
EEE	1	0.5
Saudi Digital Library	1	0.5
Others	81	38.4

**Table 3:** Students’ average duration of daily electronic resources/applications for academic performance.

Average Duration	Frequency (*n*)	Percentage (%)
Less than 1 h	30	14.2
1–2 h	42	19.9
2–3 h	65	30.8
3–4 h	29	13.7
More than 4 h	45	21.4

**Table 4:** Students’ observations of incorporation of e-learning by faculties and the age of faculties.

Incorporation of E-Learning by Faculty Members	Frequency (*n*)	Percentage (%)
More prevalent among male faculties	22	10.4
More prevalent among female faculties	33	15.6
There is no difference in the use	93	44.1
I do not know	63	29.9
Observed relation in using e-resources and age of faculty members		
More prevalent among faculties over 50 years of age	9	4.2
More prevalent among faculties under 50 years of age	103	48.8
There is no difference in use by age groups	59	28.0
I do not know	40	19.0

**Table 5:** Relationship between faculty’s dependence on social media for communication and their age.

Relationship between Faculty Member’s Dependence on Social Media and Their Age	Frequency (*n*)	Percentage (%)
More prevalent among faculties over 50 years of age	5	2.3
More prevalent among faculties under 50 years of age	103	48.8
There is no difference in use by age groups	63	29.9
I do not know	40	19.0

**Table 6:** Influence of online applications/animations on the students’ perceived academic performance.

Influence Level	Least Influence	Less Influence	Neutral Influence	More Influence	Most Influence
Scale	1	2	3	4	5
Factors					
Online presentation under 15 min	9.0	16.6	30.3	25.1	19.0
Mobile friendly	8.1	13.7	24.6	24.2	29.4
Up-to-date “look and feel” of the video	3.8	13.3	22.3	23.2	37.4
The credibility of the video	2.4	9.0	24.2	24.2	40.3
Organization/logic of the content	1.9	10.0	23.7	20.9	43.6

## Data Availability

The data presented in this study are available on request from the corresponding author. The data are not publicly available due to ethical and institutional restrictions.
